# Influence of environmental factors on the detection of blood in sheep faeces using visible–near-infrared spectroscopy as a measure of *Haemonchus contortus* infection

**DOI:** 10.1186/s13071-020-04468-6

**Published:** 2020-11-23

**Authors:** Elise A. Kho, Jill N. Fernandes, Andrew C. Kotze, Glen P. Fox, Maggy T. Sikulu-Lord, Anne M. Beasley, Stephen S. Moore, Peter J. James

**Affiliations:** 1 The Queensland Alliance for Agriculture and Food Innovation, The University of Queensland, St. Lucia, QLD 4067 Australia; 2 Commonwealth Scientific and Industrial Research Organisation (CSIRO) Agriculture and Food, St. Lucia, QLD 4067 Australia; 3 Department of Food Science and Technology, University of California, Davis, CA 95616 USA; 4 The School of Public Health, The University of Queensland, Herston, Queensland 4006 Australia; 5 The School of Agriculture & Food Sciences, The University of Queensland, Gatton, QLD 4343 Australia

**Keywords:** *Haemonchus contortus*, Visible–near infrared spectroscopy, Haemoglobin, Blood, Faecal analysis, Gastrointestinal nematodes

## Abstract

**Background:**

Existing diagnostic methods for the parasitic gastrointestinal nematode, *Haemonchus contortus*, are time consuming and require specialised expertise, limiting their utility in the field. A practical, on-farm diagnostic tool could facilitate timely treatment decisions, thereby preventing losses in production and flock welfare. We previously demonstrated the ability of visible–near-infrared (Vis–NIR) spectroscopy to detect and quantify blood in sheep faeces with high accuracy. Here we report our investigation of whether variation in sheep type and environment affect the prediction accuracy of Vis–NIR spectroscopy in quantifying blood in faeces.

**Methods:**

Visible–NIR spectra were obtained from worm-free sheep faeces collected from different environments and sheep types in South Australia (SA) and New South Wales, Australia and spiked with various sheep blood concentrations. Spectra were analysed using principal component analysis (PCA), and calibration models were built around the haemoglobin (Hb) wavelength region (387–609 nm) using partial least squares regression. Models were used to predict Hb concentrations in spiked faeces from SA and naturally infected sheep faeces from Queensland (QLD). Samples from QLD were quantified using Hemastix® test strip and FAMACHA© diagnostic test scores.

**Results:**

Principal component analysis showed that location, class of sheep and pooled versus individual samples were factors affecting the Hb predictions. The models successfully differentiated ‘healthy’ SA samples from those requiring anthelmintic treatment with moderate to good prediction accuracy (sensitivity 57–94%, specificity 44–79%). The models were not predictive for blood in the naturally infected QLD samples, which may be due in part to variability of faecal background and blood chemistry between samples, or the difference in validation methods used for blood quantification. PCA of the QLD samples, however, identified a difference between samples containing high and low quantities of blood.

**Conclusion:**

This study demonstrates the potential of Vis–NIR spectroscopy for estimating blood concentration in faeces from various types of sheep and environmental backgrounds. However, the calibration models developed here did not capture sufficient environmental variation to accurately predict Hb in faeces collected from environments different to those used in the calibration model. Consequently, it will be necessary to establish models that incorporate samples that are more representative of areas where *H. contortus* is endemic.
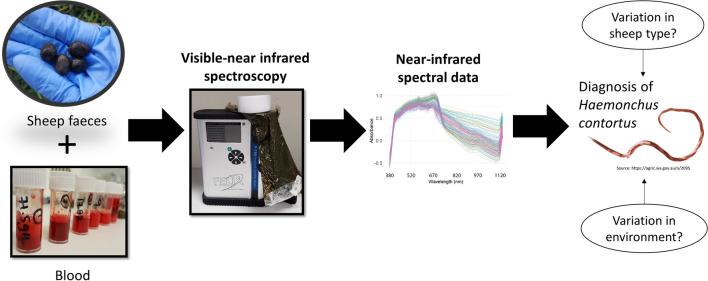

## Background

Gastrointestinal nematodes (GIN) cause significant disease and economic losses in grazing animals [1]. *Haemonchus contortus* is one of the most damaging GIN species, affecting the health and welfare of small ruminant livestock worldwide [2, 3]. Warm, moist conditions favour the survival of *H. contortus* larvae; however, larvae can also survive in relatively cold and dry conditions by suspending development in their host during winter (termed hypobiosis) and continuing their development and transmission in the following spring [4]. In Australia, *H. contortus* is most prevalent in south-eastern Queensland and northern New South Wales [5], although the distribution of *H. contortus* has been expanding to more southern parts of the country [6]. In favourable conditions, female *H. contortus* are capable of laying up to 10,000 eggs daily, resulting in rapid and extensive pasture contamination, thus increasing flock exposure to the parasite [7, 8]. Animals infected with *H. contortus* can suffer blood loss of up to 30 mL daily in severe cases [9, 10]. Blood loss in sheep typically occurs from blood ingested by the nematode and blood leakage into the sheep gut due to damage sustained through nematode attachment [10].

Slaughter of sheep to quantify nematodes is the gold standard method for the precise estimation of parasite burden in sheep. This method is also often required to demonstrate anthelmintic efficacy for the registration of new control products, but the need to euthanase sheep makes it an impractical method for use on farms [11]. Faecal worm egg count (FWEC) is the method currently recommended for GIN monitoring by the World Association for the Advancement of Veterinary Parasitology and the one used by many sheep producers [12, 13]. The most commonly used FWEC technique, the McMaster method, is known to have a relatively low analytical sensitivity of 10–50 eggs per gram of faeces (epg) and high variability between subsamples [14–17]. It has been reported that the presence of blood in faeces from infected sheep can be detected as early as 11 days after the initial infection, whereas *H. contortus* eggs begin to occur in faeces at approximately 18 days post-infection [2, 9]. Therefore, for new infections, the detection of blood or haemoglobin (Hb) in the faeces may enable earlier diagnosis of *H. contortus* infections in sheep than with FWEC. Another potential advantage of blood (haemoglobin) over eggs for diagnosis is that blood is more evenly distributed within the faeces, resulting in less variability between samples, although this needs to be confirmed.

Several tools based on blood loss have been developed for the diagnosis of *H. contortus* infections. One of these, the *Haemonchus* Dipstick Test, which is no longer commercially available, quantified the amount of faecal occult blood (FOB; blood that is not visibly apparent) present in sheep faeces [18, 19]. The FOB reagent strips Hemastix® (Bayer HealthCare LLC, Bayer AG, Leverkusen, Germany) were used to detect the presence of Hb peroxidase activity in faeces as an index of the level of worm infection present. Given that peroxidase activity can also be found in plant materials, a strict boiling time of 20 min was necessary to denature the peroxidase and ensure accuracy of the test [18, 20]. The dipstick test used a score range of 1–5, with the manufacturer’s manual stating that a score of 1 indicates a negative result and a score of 5 indicates a concentration of ≥ 200 blood cells/µL, suggesting the presence of a heavy *H. contortus* infection requiring immediate anthelmintic treatment. Detailed interpretation of the test result was challenging, however, as each score corresponded to a wide range of Hb concentrations [21]. Additionally, FOB test kits such as Hemastix® have been found to be less sensitive when used with samples from mixed-parasite infections [21].

The FAMACHA© method is a five-point scoring system that can be used in sheep yards by a trained observer to assess the level of anaemia of a given animal [22]. Relatively good sensitivity of the FAMACHA© method has been reported in various regions for detecting anaemic animals (> 50% for sheep and > 89% for goats) [23–27]. However, despite the relative ease of use and low cost of FAMACHA©, a drawback of this system is that it is relatively labour intensive and relies heavily on the operator's experience, which can lead to inconsistent results both within and across sheep management systems [23]. In addition, production losses can still occur before scores indicative of the need for anthelmintic treatment are obtained, resulting in economic losses for sheep producers [27, 29, 30]. An automated method which uses scanning of the third eyelid (palpebral conjunctiva) of the animal and image analysis to predict Hb levels has recently been validated in calves and could potentially improve the accuracy of prediction by reducing the operator variability associated with the FAMACHA© method [28]. However, whether this method is applicable to the diagnosis of helminth infections in sheep or could provide earlier prediction of the need to treat is yet to be determined.

Previous studies evaluating the accuracy of FOB test kits were performed based on the measurement of sheep blood collected by jugular venipuncture, rather than by direct measurement of FOB in faeces [11, 21]. In these studies, results from packed cell volume and Hb concentrations obtained from whole blood samples for sheep infected with *H. contortus* were compared with the predictions from the FOB test kits. It can be challenging to evaluate the accuracy of Hb assessed using FOB test kits, as there may not be a direct relationship between the changes in haematocrit levels in the host blood and the presence of blood in the faeces [21]. Furthermore, there is no quantitative method available for the direct measurement of Hb concentrations in sheep faeces.

Faecal analyses using near-infrared (NIR) spectroscopy have been previously used to evaluate diet quality in ruminants and to monitor their health and welfare [31–35]. Faecal NIR spectroscopy has also been applied to estimate tick burdens in cattle and horses through the prediction of stress [36] and internal parasite burdens through the prediction of FWEC [35]. The use of NIR spectroscopy for chemical analysis in animals has continued to increase due to its wide range of applications, low processing time, low cost, non-invasiveness, bulk-sampling capacity and the ability to measure samples under different conditions [37–39]. Importantly, it is possible to use a single NIR spectral scan of a sample to determine several chemical attributes simultaneously. However, spectroscopists must structure and design calibration models carefully to focus on specific chemical attributes related to their particular topic of interest.

The ultimate aim of a test to determine the need for anthelmintic treatment is to estimate the total worm count of *H. contortus* present in the abomasum of sheep. This is most commonly done using FWEC but, as noted, FWEC may underestimate the worm burden, given that nematodes may be present for up to 7 days before they produce eggs [40, 41]. Furthermore, methods involving the assessment of anaemia may be affected by the experience of the operator [23, 24]. Given that there are currently no tests available to directly measure sheep blood content in faeces, it is particularly challenging to determine reference values for the validation of a prediction model—in our case, the validation of a calibration model based on blood content of the faeces. For these reasons, the calibration models reported here were based on faecal samples spiked with known concentrations of sheep blood.

We previously reported the potential of visible (Vis)–NIR spectroscopy for detecting blood in sheep faeces, as an indicator of *H. contortus* infection [42]. In that study, we developed calibration models within the wavelength region of 400–600 nm to measure blood in faeces collected from a single sheep and found that the prediction precision was lowest with the lower Hb concentrations, likely due to the lack of physical, chemical and environmental variation in the faecal samples in the calibration model. In the study reported here, we investigated the predictability of calibration models built using faeces collected from various locations free from *H. contortus* and spiked with known amounts of blood, for the prediction of Hb concentrations in both blood-spiked and naturally infected sheep faeces.

## Methods

### Faecal sample collection and faeces–blood preparation

In order to obtain blood-free field-collected sheep faeces from a range of different environments and sheep backgrounds, faecal samples were collected during the winter–spring of 2019 (August–October) from multiple sheep properties in South Australia (SA) known to be free from *H. contortus* infection (based on previous worm egg counts and larval differentiations). Faecal samples were collected both as individual samples from single sheep and as pooled samples where ten individual samples of faeces were combined and later subsampled for Vis–NIR analysis. Faecal consistency was described as very dry pellets (VDP), dry pellets (DP), dry aggregates (DA) moist pellets (MP) and very moist pellets (VMP). Fresh samples were collected off the ground from ewes, rams, lambs (< 1 year old) and hoggets (1–2 years old) into separate sample bags shortly after defecation. Faecal samples were transported on ice to Brisbane, QLD and stored at − 20 °C until further analysis. Faecal samples were also collected from Armidale, New South Wales (NSW) as described in Kho et al. [42]. These samples were collected from a single uninfected sheep kept in an animal house, fed a diet consisting of ground wheat grain and ground lucerne hay. For the purpose of this study, samples from this animal are defined as the uninfected control samples. All animal procedures were approved by the FD McMaster Animal Ethics Committee, CSIRO Agriculture and Food (Animal Ethics Approval Number AEC 18/09).

The frozen samples were thawed over a 2-h period, and the weights of faeces from each sample were recorded. Faeces from each collection bag was thoroughly homogenised, and 10-g subsamples were mixed with various concentrations of diluted defibrinated sheep blood. Faeces–blood samples and controls were sandwiched between two sheets of polyethylene film (Woolworths Australia, Bella Vista, NSW, Australia) and thoroughly dispersed by rolling and folding each sample multiple times to form a ‘slab’ (10 × 20 cm) before measurement at randomly selected points with a Vis–NIR spectrometer [43].

Defibrinated sheep blood used throughout the study was purchased from a commercial supplier (Serum Australis, Manilla, NSW, Australia) and serially diluted with distilled water at the ratio of 1:2. Each slab consisted of 10 g of faeces mixed with 5 mL of diluted blood to provide final concentrations of 0, 2, 2.3, 4, 4.25, 4.5, 8, 8.5 and 9 µg Hb/mg faeces. Control samples were prepared by adding 5 mL water to 10 g faeces.

Faeces were also collected from The University of Queensland (UQ) Gatton campus, QLD, where *H. contortus* is endemic, on 2 days in February and March 2020, respectively. A total of 221 mm of rainfall had been recorded over the preceding 2 months, providing favourable conditions for *H. contortus* transmission [44, 45]. These samples were included to represent samples collected from a different region known to be prone to *H. contortus* infections. Sheep faeces were collected off the ground shortly after defecation and were placed into individual sample bags. Sheep identification (ID) was recorded on all collection bags. The FAMACHA© scores and history of anthelmintic treatments for the corresponding sheep ID were obtained and recorded on the same day as faeces collection by trained and experienced veterinarians at UQ Gatton, QLD. Faecal samples from each bag were homogenised and subsamples processed within 24 h of collection using the McMaster method to determine FWEC and Hemastix® to estimate the amount of blood present in the faeces. The remainder of each sample was stored at − 20 °C until further analysis with Vis–NIR spectroscopy. Prior to spectroscopic measurement, the faecal samples were thawed for 2 h and later scanned as a slab (10 × 20 cm) sandwiched between two sheets of polyethylene film. All animal procedures here were approved by the UQ Animal ethics committee (Animal Ethics Approval Number AEC: SVS/452/17).

### Calibration and validation dataset

As the diagnosis of *H. contortus* infection using FWEC and Hemastix® is typically performed by pooling faeces collected from multiple individual animals [18, 46], we determined the effects of using pooled or individual samples for building calibration models. Two calibration models were built using faeces collected from different locations in SA and NSW. Model 1 consisted of faeces from individual sheep, while Model 2 included faeces from both individual and pooled samples. Table 1 shows the description of samples included in each model.

**Table 1:** Description of samples included in the calibration models built for prediction of haemoglobin in sheep faeces using a visible–near-infrared spectrometer

Model	Sample origin	Sample type	Class of sheep	*n*
1	Adelaide Hills, SA	Individual	Ewes	44
	Armidale, NSW	Single animal	Wether (uninfected control)	6
	Fleurieu Peninsula, SA	Individual	Ewes and lambs	24
	Currency Creek, SA	Individual	Ewes	10
	Eyre Peninsula, SA	Individual	Ewes and hoggets	35
	Total			119
2	Adelaide Hills, SA	Individual	Ewes	44
	Armidale, NSW	Single animal	Wether (uninfected control)	6
	Mid-North 1, SA	Pooled	Ewes	22
	Mid-North 2, SA	Pooled	Ewes	13
	Fleurieu Peninsula, SA	Individual	Ewes and lambs	24
	Currency Creek, SA	Individual	Ewes	10
	Eyre Peninsula, SA	Individual	Ewes and hoggets	35
	Total			153

*NSW* New South Wales, *SA* South Australia

Four groups of samples were prepared and measured to form the validation datasets. The Hb concentrations of the faecal samples used in the validation datasets were 0, 2, 2.3, 4, 4.25, 4.5, 8, 8.5 and 9 µg Hb/mg faeces. For samples collected as ‘individual’ sample type, each bag of faeces was subsampled, spiked with blood and scanned as individual samples for each Hb concentrations. For samples designated as ‘pooled’, replicates of each faeces were subsampled and spiked with blood before scanning using Vis–NIR spectrometer. To include variations that may be present in future validations, a randomly selected portion of faecal samples included in Models 1 and 2 were mixed with various concentrations of Hb and scanned as a slab on separate days using the Vis–NIR spectrometer (Val1 and Val2). Faecal samples were collected from the Lower North and Yorke Peninsula in SA to represent locations not included in the models as validation dataset 3 (Val3). Faeces were also collected from UQ Gatton, QLD as validation dataset 4 (Val4) to represent samples from different regions with naturally occurring *H. contortus* infections. Table 2 shows detailed descriptions of the validation datasets used in this study.

**Table 2:** Description of samples included in the validation datasets

Validation dataset	Sample origin	Sample type	Class of sheep	*n*
Val1	Mid-North 1, SA	Pooled	Ewes	7
	Mid-North 2, SA	Pooled	Ewes	5
	Adelaide Hills, SA	Individual	Ewes	23
	Armidale, NSW	Single animal	Wether (uninfected control)	4
	Total			39
Val2	Eyre Peninsula, SA	Individual	Ewes and hoggets	32
	Armidale, NSW	Single animal	Wether (uninfected control)	3
	Total			35
Val3	Lower North, SA	Pooled	Ewes and rams	22
	Yorke Peninsula, SA	Individual	Ewes	10
	Total			32
Val4	Gatton, QLD	Individual	Ewes	22
	Total			22

### Spectral acquisition with Vis–NIR spectroscopy

A Felix F-750 portable Vis–NIR spectrometer (Felix Instruments, Camas, WA, USA), equipped with a xenon tungsten lamp as the light source, was used to collect spectra throughout this study. The Vis–NIR spectra of blood in sheep faeces were obtained within the wavelength range of 300–1200 nm at a spectral resolution of between 8 and 13 nm and data resolution of 3 nm. Each spectrum was obtained using 32 scans. Faeces–blood mixture slabs were placed on top of the scanning window of the Felix F-750 spectrometer, and a white Teflon disc was placed above the slab to augment the signal-to-noise ratio [42]. Spectra were acquired randomly between prepared faeces slabs and across ten random scanning points on each faeces–blood mixture slab using a randomised list generated from Microsoft Excel (2016 version; Microsoft Corp., Redmond, WA, USA). The average of ten spectra from each slab was calculated and used for the development of the Vis–NIR calibration models.

### Faecal worm egg count, Hemastix® and FAMACHA©

Faecal worm egg counts were performed using the modified McMaster method [12]. Briefly, faeces from each collection bag were homogenised, and a subsample of 2 g faeces was mixed with 16 mL of concentrated salt solution (MgSO_4_, specific gravity [SG] = 1.18). The faecal slurry was strained through a tea strainer to remove large particulate matter, and a subsample of the egg suspension was examined by microscopy using the 0.3-mL McMaster counting chamber, giving a lower detection level of 30 epg.

As the faecal samples collected from QLD were naturally infected with *H. contortus*, the blood content in the faeces was unknown. Furthermore, there was no available method to accurately measure the blood concentrations in the faeces. Therefore, the amount of faecal occult blood in the faeces from the QLD samples was estimated using Hemastix® (Bayer Australia, Pymble, NSW, Australia) based on a modified protocol described by Colditz and Le Jambre [18]. Briefly, two subsamples from each sample bag were diluted in water to yield a final dilution of 1:500. Samples were boiled for 20 min and cooled for 3–5 min in a container with tap water before testing. Hemastix® scores were obtained by dipping the reagent strips into the cooled diluted faecal mixture, and the colour change of the strip was assessed against a reference colour chart (provided on the reagent bottle) after 60 s to provide a score between 1 (no color change) and 5 (dark green).

The FAMACHA© scores of individual sheep were obtained by examining the colour of the lower eyelid mucous membranes and comparing it to a chart of colour standards, with a score of 1–2 representing ‘not anaemic’ and scores of 3–5 indicating levels of anaemia requiring treatment [22]. Of the 22 samples collected from Gatton, QLD, eight were collected at random with no prior knowledge of infection history or FAMACHA© score; thus, these samples were removed from the analysis. The Vis–NIR-predicted Hb values for the faecal samples were then compared with the results from the FWEC, Hemastix® and FAMACHA© assessments.

### Chemometric analysis and statistics

All spectral data were exported using F750 DataViewer (v 1.2) and analysed as raw spectra using The Unscrambler X (v. 10.5.1; CAMO A/S, Oslo, Norway). RStudio (v. 1.0.153; https://rstudio.com/products/rstudio/) was used to establish the plots presented here. The spectral data were pre-treated using the Savitzky–Golay filter with second derivative order smoothing, second polynomial order and seven smoothing points to remove scattering effects from the spectra prior to further analysis. Spectral measurements were initially investigated using principal component analysis (PCA) to identify spectrally similar samples, and outliers were identified using the Hotelling *T*^2^ statistics. Spectra that had extreme spectral signals, leverage and residual means relative to other samples were considered to be outliers. The impact of sample origin (location), class of sheep, faecal consistencies and sample type on the Savitzky–Golay transformed spectra was analysed using PCA within the wavelength range of 387–609 nm.

The calibration models for the detection of Hb in sheep faeces were developed using partial least squares (PLS) regression analysis with randomised blocks of cross-validation. Calibration models built in this study were confined within the wavelength range of 387–609 nm, which contained the relevant Hb absorption bands [42]. The coefficient of determination for correlation in calibration (${R^{2}}_{{{\text{cal}}}}$), root-mean-square error of calibration (RMSEC), coefficient of determination for correlation in cross-validation (${r^{2}}_{{{\text{cv}}}}$) and the root-mean-square error of cross-validation (RMSECV) were used to assess the model performance. The number of latent variables (LVs) was determined by the leave-one-out cross-validation method [47–49]. For analysis, FWEC was transformed using log_10_(FWEC + 10). A transformed epg of 2.82 (650 epg) was used as the threshold for anthelmintic treatment [43]. Prediction statistics of the validation dataset was evaluated using the coefficient of determination for correlation of prediction (${r^{2}}_{{\text{p}}}$), and root-mean-squared error of prediction (RMSEP). Predictions are considered good if they have ${r^{2}}_{{\text{p}}} > 0.80$ with the smallest RMSEP. A level of 3 µg Hb/mg faeces was used as a threshold for anthelmintic treatment to calculate the prediction accuracies for the calibration models built in this study [42, 50]. The sensitivity (%SN) of the model refers to the percentage of samples correctly identified as indicating the need for treatment (> 3 µg Hb/mg faeces), while the specificity (%SP) of the model refers to the percentage of the samples correctly predicted as ‘healthy’ samples where treatment was not needed (< 3 µg Hb/mg faeces). The formulas used for the calculation of sensitivity and specificity of the predicted results are as follows:

$$\% {\text{SN}} = \frac{{{\text{True}}\;{\text{positive}}}}{{{\text{True}}\;{\text{positive}} + {\text{False}}\;{\text{negative}}}},$$

$$\% {\text{SP}} = \frac{{{\text{True}}\;{\text{negative}}}}{{{\text{False}}\;{\text{positive}} + {\text{True}}\;{\text{negative}}}}.$$

True positive indicates samples containing > 3 µg Hb/mg faeces that were correctly predicted, whereas true negative indicates samples containing < 3 µg Hb/mg faeces that were correctly predicted.

PCA was also applied to the QLD samples for analysis of the pre-processed spectra (Savitzky–Golay 1st derivative, 2nd polynomial order and 5 smoothing points) within the wavelength region of 387–609 nm for analysis of the loading and score plots.

## Results

**Fig. 1:**
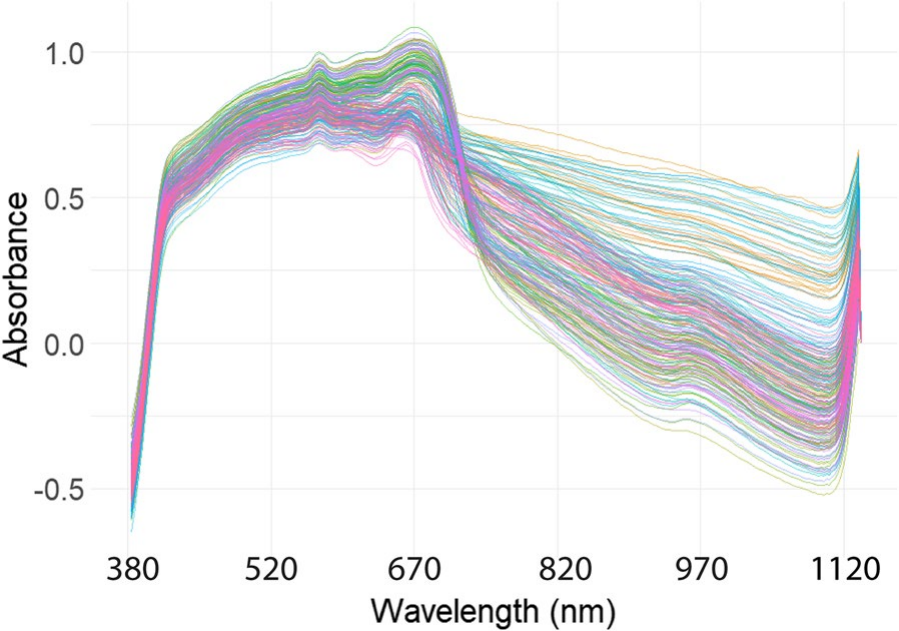
Raw absorbance visible–near infrared spectra of all faeces–blood mixture samples prepared for the prediction of haemoglobin in sheep faeces

The raw mean-centred absorbance spectra of sheep faeces containing various Hb concentrations covering the range of 380–1200 nm are illustrated in Fig. 1. All faecal samples collected in this study showed a similar profile and trend. Two distinct bands were observed around 576 and 670 nm, which had been found previously to be associated with Hb and chlorophyll, respectively [42, 51, 52]. Subsequently, for the prediction of Hb in sheep faeces in the present study, all analyses were carried out within the wavelength range of 387–609 nm to limit the effects of the 670-nm chlorophyll peak.

**Fig. 2:**
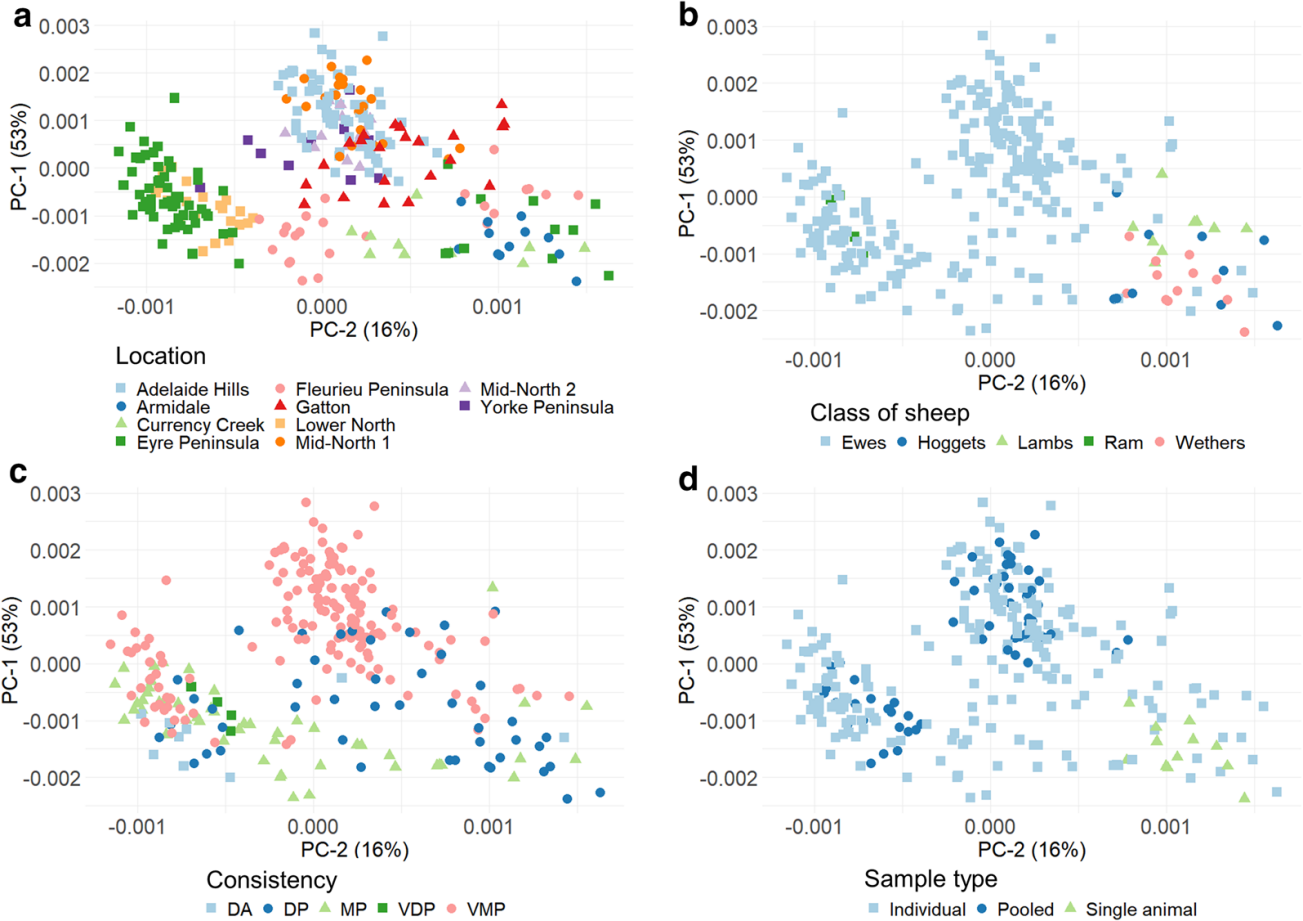
Score plots of principal component analysis (PCA) for Savitzky–Golay filtered spectra of all samples measured using visible–near infrared (Vis–NIR) spectrometry within the wavelength region of 387–609 nm. Samples are grouped by location (**a**), class of sheep (**b**), faeces consistency (**c**) and sample type (**d**).* DA* Dry aggregates,* DP* dry pellets, *MP* moist pellets,* PC-1*, *PC-2* principal components 1, 2, respectively,* VDP* very dry pellets,* VMP* very moist pellets

PCA of the Savitzky–Golay filtered spectra indicated that the first two principal components, PC-1 and PC-2, explained 53 and 16% of the total variance, respectively (Fig. 2). The samples appeared to primarily cluster on the basis of the location from which the faeces were collected (Fig. 2a). Samples collected from Mid-North 1 and 2 and Adelaide Hills were spectrally similar and clustered around the positive values of PC-1. Samples from the Eyre Peninsula were split into two clusters, with a majority of the samples lying in the quadrant of positive values of PC-2 along with samples from Lower North, while the remaining samples clustered in the negative values of PC-2. The separation between samples from Eyre Peninsula was associated with the difference in class of sheep; samples in the positive values of PC-2 were associated with samples collected from ewes, while the remaining samples were collected from hoggets (Fig. 2b). Samples from the Fleurieu Peninsula also showed clustering; samples collected from ewes were clustered within the positive quadrant of PC-2, while samples collected from lambs clustered within the negative quadrant of PC-2.

Although no apparent clustering was observed when faecal samples were grouped based on the consistency of the faeces (Fig. 2c), it was found that the pooled samples clustered more closely together (Fig. 2d). In contrast, individual samples were more scattered (Fig. 2d).

Table 3 shows the prediction accuracies for the models built using PLS regression with the Savitzky-Golay-transformed spectra. The two models showed similar performance statistics of ${R^{2}}_{{{\text{cal}}}}$ = 0.48–0.49 and RMSEC = 2.26–2.27 µg Hb/mg faeces for the prediction of Hb concentrations in field-collected sheep faeces. The inclusion of pooled samples in Model 2 gave a slight improvement in the performance for both calibration and cross-validation compared with Model 1, which consisted of individual samples. Still, this difference is unlikely to be significant.

**Table 3:** Prediction statistics for the calibration models built with Savitzky–Golay transformed spectra of faeces with various haemoglobin concentrations using the wavelength region of 387–609 nm

Calibration		Regression analysis	Latent variables (*N*)	Calibration		Cross-validation	
Models	*N*			${R^{2}}_{{{\text{cal}}}}$	RMSEC	${r^{2}}_{{{\text{cv}}}}$	RMSECV
1	119	PLS	6	0.48	2.27	0.31	2.65
2	153	PLS	6	0.49	2.26	0.33	2.59

PLS, Partial least squares; ${R^{2}}_{{{\text{cal}}}}$, coefficient of determination for correlation in calibration; RMSEC, root-mean-squared error of calibration (units in µg Hb/mg faeces); ${r^{2}}_{{{\text{cv}}}}$, coefficient of determination for correlation in cross-validation; RMSECV, root-mean-squared error of cross-validation (units in µg Hb/mg faeces)

The calibration models were applied to the validation datasets to predict Hb in faecal samples collected from various locations. Table 4 shows the prediction accuracies for validation datasets 1–3. Prediction of Hb in a mixture of pooled and individual samples collected from Mid-North 1 and 2, Adelaide Hills and Armidale (Val1) showed poor prediction statistics (${r^{2}}_{{\text{p}}}$ < 0.05 and RMSEP > 2.78 µg Hb/mg faeces) using both Model 1 and Model 2. In contrast, predictions of Hb in both pooled and individual faecal samples collected from Eyre Peninsula, Armidale, Lower North and Yorke Peninsula (Val2 and Val3) showed average prediction statistics (${r^{2}}_{{\text{p}}}$ > 0.6 and RMSEP = 1.53–1.73 µg Hb/mg faeces) using both models. Based on a threshold for treatment of 3 µg Hb/mg faeces, high sensitivity was observed for Val1 when Model 1 and 2 were employed to predict for high Hb concentrations (> 3 µg Hb/mg faeces) in the faecal samples (%SN > 71.4%). However, there was low specificity (%SP < 66.7%) for samples with low Hb concentrations (< 3 µg Hb/mg faeces). The overall sensitivity and specificity of Hb predictions were higher for Val2 (%SN > 82.5%, %SP > 72.2%) and Val3 (%SN > 88.9%, %SP > 64.3%) than for Val1, and Hb predictions performed with higher accuracies using Model 2 than Model1 (Table 4).

**Table 4:** Performance of Models 1 and 2 for Savitzky–Golay transformed spectra of faecal samples from validation dataset Val1, Val2 and Val3 for the prediction of haemoglobin in faeces collected from South Australia within the wavelength range of 387–609 nm

Model	Validation dataset^a^											
	Val1 (*n* = 39)				Val2 (*n* = 35)				Val3 (*n* = 33)			
	${r^{2}}_{{\text{p}}}$	RMSEP (µg Hb/mg faeces)	%SN (95% CI)	%SP (95% CI)	${r^{2}}_{{\text{p}}}$	RMSEP (µg Hb/mg faeces)	%SN (95% CI)	%SP (95% CI)	${r^{2}}_{{\text{p}}}$	RMSEP (µg Hb/mg faeces)	%SN (95% CI)	%SP (95% CI)
1	0.05	2.78	71.4% (47.8–88.7%)	44.4% (21.5–69.2%)	0.73	1.57	88.2% (63.6–98.5%)	72.2% (46.5–90.3%)	0.77	1.67	88.9% (65.3–98.6%)	64.3% (35.1–87.2%)
2	NA	3.03	57.1% (34.0–78.2%)	66.7% (41.0–86.7%)	0.68	1.73	82.4% (56.6–96.2%)	77.8% (52.4–93.6%)	0.76	1.73	94.4% (72.7–99.9%)	78.6% (49.2–95.3%)

${r^{2}}_{{\text{p}}}$, Coefficient of determination for correlation of prediction; RMSEP, root-mean-squared error of prediction (units in µg Hb/mg faeces); %SN (95% CI): sensitivity of the model with a 95% confidence interval; %SP (95% CI), specificity of the model with a 95% CI

^a^To include variations that may be present in future validations, a randomly selected portion of faecal samples included in Models 1 and 2 were mixed with various concentrations of haemoglobin and scanned as a slab on separate days using the visual–near-infrared spectrometer (Val1 and Val2). Faecal samples were collected from the Lower North and Yorke Peninsula in South Australia to represent locations not included in the models as validation dataset 3 (Val3)

**Fig. 3:**
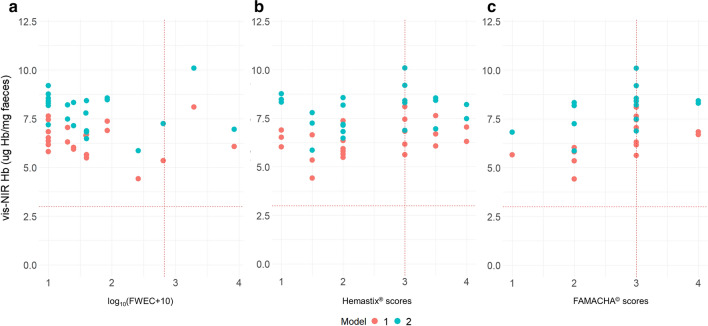
Relationship between the predicted haemoglobin (*Hb*) using Vis–NIR spectroscopy in faecal samples collected from Gatton, Queensland (QLD) and log_10_(faecal worm egg count [*FWEC*] + 10) (**a**), Hemastix® scores (**b**) and FAMACHA© scores (**c**). The red dotted lines represent the cutoff points for anthelmintic treatment for the different methods

Figure 3 shows the relationship between Hb concentrations predicted using Vis–NIR spectroscopy (Models 1 and 2) and log_10_(FWEC + 10), Hemastix® and FAMACHA© for samples collected from QLD. The FWEC for samples collected from QLD contained between 0 epg (transformed epg = 0) and 8340 epg (transformed epg = 3.93), while the blood leakage levels as indicated by Hemastix® and FAMACHA© ranged from 1 to 4, respectively. Overall, high levels of Hb were predicted for all QLD samples using the Vis–NIR spectroscopy (> 4 µg Hb/mg faeces), including samples found to contain low blood leakage based on Hemastix® and FAMACHA© tests. On average, the Vis–NIR-predicted Hb values in QLD faeces were slightly lower using calibration Model 1 than Model 2. As with the samples from SA, faecal consistency did not appear to affect the Hb predictions for the faeces collected from QLD.

**Fig. 4:**
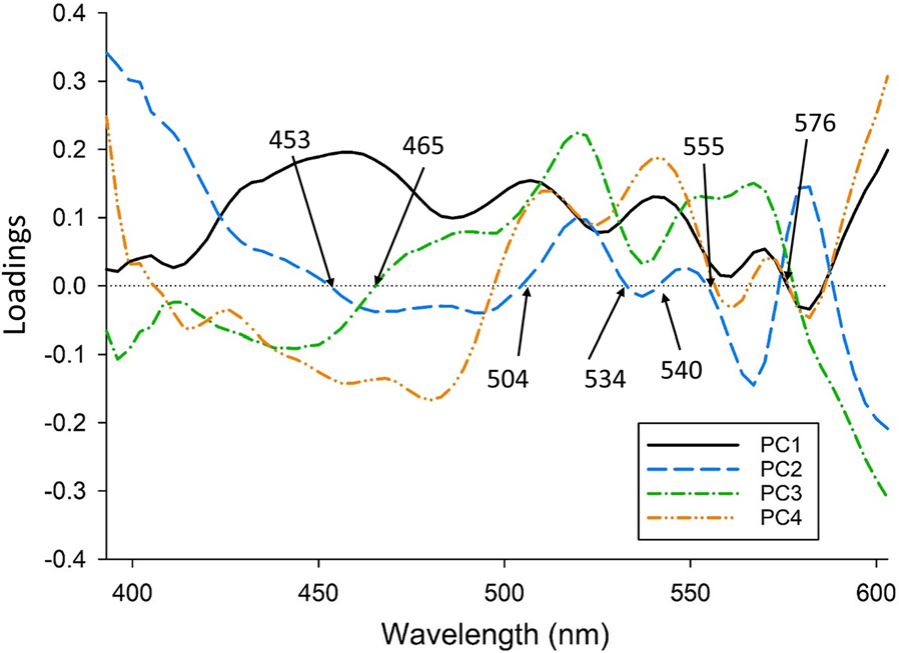
PCA loading plot of the Savitzky–Golay (1st derivative order, 2nd polynomial order and 5 smoothing points) within the wavelength range of 387–609 nm for sheep faeces naturally infected with *Haemonchus contortus* collected from Queensland.* PC1*–*PC4* Principal components 1–4

Figure 4 shows the PCA loading plot for Savitzky–Golay pre-processed spectra of the samples collected from QLD. Bands of interest for spectra pre-processed with Savitzky–Golay first derivative typically lie in the zero plane of the loading units [53]. The first four principal components (PCs) accounted for 96% of the total spectral variation observed. Based on the PCA loading plots, the peaks at 453, 465, 504, 534, 540, 555 and 576 nm contributed to the majority of the spectral variation in the naturally infected QLD samples.

**Fig. 5:**
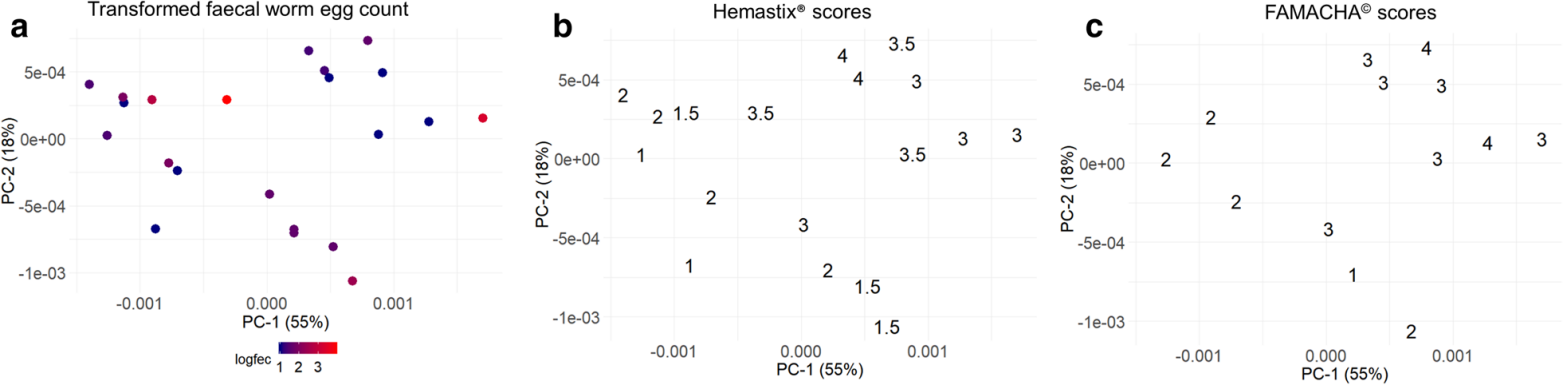
Score plots of PC-1 and PC-2 from the PCA performed within the region of 387–609 nm for the visible–near-infrared spectra of faecal samples collected from Gatton, QLD. Samples were pre-processed using Savitzky–Golay derivative smoothing and grouped based on log_10_(faecal worm egg count + 10) (**a**), Hemastix® scores (**b**) and FAMACHA© scores (**c**)

Interestingly, the PCA score plots of the pre-processed spectra for all of the naturally infected QLD samples showed clustering based on their Hemastix® and FAMACHA© scores (Fig. 5). The samples with high Hemastix® and FAMACHA© scores (> 3), indicating that anthelmintic treatment may be required, clustered in the positive quadrant of PC-1, with one potential outlier. The remaining samples with lower scores trended from the positive to the negative quadrant of PC-2.

## Discussion

The development of a more effective and rapid approach for diagnosing *H. contortus* infections would have significant benefits for sheep producers. Accurate detection of the presence of Hb in faeces may also enable earlier diagnosis of *H. contortus* infection in sheep than is possible using existing methods as blood can be detected in the faeces approximately 7 days before *H. contortus* eggs are found [9].

In this study, the Vis–NIR models for sheep blood content successfully differentiated ‘healthy’ samples from those requiring anthelmintic treatment with moderate to good prediction accuracy (sensitivity 57–94%, specificity 44–79%). However, PCA indicated that the pre-processed spectra were clustered on the basis of location and the class of sheep from which the samples were collected. It has previously been demonstrated that faeces obtained from animals grazing on different rangelands and pastures vary in a number of components, including long-chain alcohols, very-long-chain fatty acids and alkanes [54, 55], all of which will affect the absorbance of the spectra. Therefore, the separation of samples from different locations may reflect the diet consumed by the sheep, as different locations are likely to be associated with differing pasture compositions.

Differences in the faecal Vis–NIR spectra in different classes of sheep have previously been reported by Godfrey et al. [56] who found differences between male, pregnant and lactating animals [56]. The differences in the faecal Vis–NIR spectra in age classes of sheep seen in our study are not surprising and may be related to differences in maturity of the digestive systems between younger and older sheep, which have been previously shown to affect the faecal NIR spectra from goats [57]. No clear separation was seen due to the faecal consistency (Fig. 2c), which suggests that the moisture of the faecal samples did not affect the prediction accuracy of models established using the wavelength region of 387–609 nm. This is consistent with the results from our previous study which showed that Hb concentrations in sheep faeces were accurately predicted regardless of whether the samples were dried or not prior to scanning [42]. Although samples prepared for NIR spectroscopy often require drying to avoid interference from moisture [38, 39], our results suggest that moisture content does not affect the prediction of Hb concentrations in sheep, eliminating the need to dry faecal samples for on-farm application.

Pooled faecal samples clustered more closely together than individual samples (Fig. 2d). These samples were thoroughly homogenised before adding blood to the faeces, resulting in an evenly mixed faeces–blood mixture with minimal variation between samples. The use of pooled faecal samples for faecal NIR analysis has also been recommended for the prediction of diet qualities in livestock [58] as the nutritional diet backgrounds of animals grazing in the same area are similar enough to provide a level of precision adequate for herd management [59]. Our results show that the inclusion of pooled samples in the calibration model did not significantly affect the prediction statistics of the prediction models. This result has important implications, because pooled sample collection is far more practical than the use of individual samples for parasite surveillance in large sheep flocks, and is also the recommended method for sampling FWEC to determine the need for flock anthelmintic treatments [12]. The inclusion of a mixture of pooled and individual samples in a calibration model will assist in capturing the majority of the variations observed between individuals in a mob of sheep, thus improving the robustness of the prediction models developed.

The Hb predictions for samples in Val1 showed lower accuracy than the other datasets. Samples in Val1 were freeze–thawed multiple times, and while it is not known how the freeze–thaw process affects the chemical composition of faeces, we have previously observed difference in spectra between samples stored at 4 °C and those stored at − 20 °C (unpublished data), suggesting that the temperature may have been a factor. In contrast, the Hb for samples in Val2 were predicted with a high level of accuracy using models established with both individual (Model 1) and mixed pooled and individual samples (Model 2). Although a higher ${r^{2}}_{{\text{p}}}$ and lower RMSEP prediction statistics with Model 1 indicated slightly better Hb predictions for samples in Val2 compared to Model 2, the sensitivity and specificity of the predicted Hb were higher for these samples with Model 2 (mixed pooled and individual samples) than Model 1. Similarly, sensitivity and specificity based on a threshold for treatment at 3 µg Hb/mg faeces were higher for the prediction of Hb in samples from Val3 using Model 1 than with Model 2. This suggests that the inclusion of pooled samples in the calibration model may improve prediction of the need for anthelmintic treatment, which is in line with the recommendations provided for using faecal NIR spectroscopy to estimate nutritional profiles in livestock [58].

Although the locations of origin of faecal samples in Val3 (Lower North and Yorke Peninsula) were not represented in either of the calibration models, high accuracies for the prediction of Hb were observed for these samples using both Model 1 and 2. This may be due in part to similarities in faecal composition of samples, as these samples originate from similar climatic zones of SA with similar sheep management and likely similar pasture compositions between the two areas.

In contrast, validation using samples collected from QLD did not show any relationship with FWEC, Hemastix® scores and FAMACHA© scores (Fig. 3) and all samples collected from QLD were predicted to have high Hb concentrations. This may be due to a number of factors. The low precision may have been related to the difference in faecal composition between the samples collected in SA and QLD, which may be due in part to differences in climate or the pastures on which the sheep were grazing. Additionally, the calibration models were built based on spiked samples from SA for which the concentration of blood was accurately known. The amount of blood in the faeces from QLD samples was unknown and estimated using Hemastix® and FAMACHA© scores. Thirdly, of the peaks observed from the PCA loading plots for QLD samples, only two peaks had been previously found to be associated with blood. The two peaks, at 540 and 576 nm, respectively, which are associated with oxyhemoglobin [60, 61], were observed in our study with spiked blood [42]. In contrast, the peak at 555 nm was not previously observed and has been shown to be due to deoxyhemoglobin [60]. Therefore, the lower precision for the prediction of Hb in QLD samples could also be due in part to the difference in blood chemicals between naturally occurring FOB and the blood used to spike the faeces in the calibration models.

Interestingly, when PCA was performed on the pre-processed spectra, we found that the QLD faecal samples showed separation between samples with high Hemastix® and FAMACHA© scores (> 3) and those with lower scores. This result confirms that the collected Vis–NIR spectra within the wavelength range of 387–609 nm for QLD samples were correlated at some level to blood loss found in the faecal samples.

The results from PCA did not show any clear relationship between Vis–NIR-predicted Hb concentrations and the transformed FWEC, which may be due to the fact that *H. contortus* eggs are typically detected around 1 week after the presence of blood in sheep faeces [18, 62] and may not have been present in some or all of the sheep at the time of sampling. Results from this experiment suggest that further research to expand the calibration model by including faecal samples collected from a range of areas in QLD that are naturally infected with *H. contortus* is necessary.

## Conclusion

The calibration models built in this study using faeces spiked with sheep blood were useful in predicting Hb for faecal samples within the same geographical region and provided further evidence for the feasibility of using Vis–NIR spectroscopy for the diagnosis of *H. contortus* infections in sheep. The location where the faecal samples were collected, the class of sheep and the type of faecal samples (pooled or individual) were significant factors affecting the predictability of the calibration model. The prediction sensitivity and specificity based on the anthelmintic treatment threshold (3 µg Hb/mg faeces) for both pooled and individual samples were high, particularly when using a calibration model built on a mixture of pooled and individual animals. Although the models built using blood-spiked faecal samples from sheep in SA were not predictive for samples collected from QLD, we demonstrated that Vis–NIR spectroscopy shows high potential for determining which sheep or mob of sheep require treatment. Furthermore, this is the first study to identify the peaks within the visible range for FOB found in naturally infected sheep faeces, which will provide substantial insight for the future development of Vis–NIR models for predicting FOB. To further develop a field-ready Vis–NIR calibration model, future work should focus on using faeces collected from naturally infected animals across a wide range of environments and sheep types in *Haemonchus*-endemic areas.

## Data Availability

Not applicable.

## Abbreviations

DA: Dry aggregate
DP: Dry pellet
epg: Eggs per gram
FOB: Faecal occult blood
FWEC: Faecal worm egg count
GIN: Gastrointestinal nematode
Hb: Haemoglobin
LVs: Latent variables
MP: Moist pellet
NIR: Near-infrared
NSW: New South Wales
PCA: Principal component analysis
PC: Principal component
PLS: Partial least squares
${R^{2}}_{{{\text{cal}}}}$: Coefficient of determination for correlation in calibration
QLD: Queensland
RMSEC: Root-mean-square error of calibration
${r^{2}}_{{{\text{cv}}}}$: Coefficient of determination for correlation in cross-validation
RMSECV: Root-mean-square error of cross-validation
${r^{2}}_{{{\text{p}}}}$: Coefficient of determination for correlation of prediction
RMSEP: Root-mean-squared error of prediction
SA: South Australia
VDP: Very dry pellet
VMP: Very moist pellet
Vis–NIR: Visible–near-infrared
%SN: Sensitivity in percentage
%SP: Specificity in percentage
